# An Efficient Optimization Method for Solving Unsupervised Data Classification Problems

**DOI:** 10.1155/2015/802754

**Published:** 2015-07-29

**Authors:** Parvaneh Shabanzadeh, Rubiyah Yusof

**Affiliations:** ^1^Centre for Artificial Intelligence and Robotics, Universiti Teknologi Malaysia, 54100 Kuala Lumpur, Malaysia; ^2^Malaysia-Japan International Institute of Technology (MJIIT), Universiti Teknologi Malaysia, 54100 Kuala Lumpur, Malaysia

## Abstract

Unsupervised data classification (or clustering) analysis is one of the most useful tools and a descriptive task in data mining that seeks to classify homogeneous groups of objects based on similarity and is used in many medical disciplines and various applications. In general, there is no single algorithm that is suitable for all types of data, conditions, and applications. Each algorithm has its own advantages, limitations, and deficiencies. Hence, research for novel and effective approaches for unsupervised data classification is still active. In this paper a heuristic algorithm, Biogeography-Based Optimization (BBO) algorithm, was adapted for data clustering problems by modifying the main operators of BBO algorithm, which is inspired from the natural biogeography distribution of different species. Similar to other population-based algorithms, BBO algorithm starts with an initial population of candidate solutions to an optimization problem and an objective function that is calculated for them. To evaluate the performance of the proposed algorithm assessment was carried on six medical and real life datasets and was compared with eight well known and recent unsupervised data classification algorithms. Numerical results demonstrate that the proposed evolutionary optimization algorithm is efficient for unsupervised data classification.

## 1. Introduction

Unsupervised data classification (or data clustering) is one of the most important and popular data analysis techniques and refers to the process of grouping a set of data objects into clusters, in which the data of a cluster must have high degree of similarity and the data of different clusters must have high degree of dissimilarity [1]. The aim is to minimize the intercluster distance and maximize the intracluster distance. Clustering techniques have been applied in many areas such as document clustering [2, 3], medicine [4, 5], biology [6], agriculture [7], marketing and consumer analysis [8, 9], geophysics [10], prediction [11], image processing [12–14], security and crime detection [15], and anomaly detection [16].

In clustering problem, a dataset is divided into *k* number of subgroups such that elements in one group are more similar to one another than elements of another group [17]. It can be defined to find out unknown patterns, knowledge, and information from a given dataset *A* which was previously undiscovered using some criterion function [18]. It is NP complete problem when the number of cluster is greater than three [17]. Over the last two decades, many heuristic algorithms have been suggested and it is demonstrated that such algorithms are suitable for solving clustering problems in large datasets. For instance, the Tabu Search Algorithm for the clustering is presented in [19], the Simulated Annealing Algorithm in [20], the Genetic Algorithm in [21], and the particle swarm optimization algorithm in [22], which is one of powerful optimization methods. Fernández Martínez and Garcia-Gonzalo [23–26] clearly explained how PSO family parameters should be chosen close to the second order stability region. Hatamlou et al. in [27] introduced the Big Bang Big Crunch algorithm for the clustering problem. This algorithm has its origin from one of the theories of the evolution of the universe, namely, the Big Bang and Big Crunch theory. An Ant Colony Optimization was developed to solve the clustering problem in [28]. Such algorithms are able to find the global solution to the clustering. Application of the Gravitational Search Algorithm (GSA) [29] for clustering problem has been introduced in [30]. A comprehensive review on clustering algorithms can be found in [31–33].

In this paper, a new heuristic clustering algorithm is developed. It is based on the evolutionary method called the Biogeography-Based Optimization (BBO) method proposed in [34]. The BBO method is inspired from the science of biogeography; it is a population-based evolutionary algorithm. Convergence results for this method and its practical applications can be found in [35]. The algorithm has demonstrated good performance on various optimization benchmark problems [36]. The proposed clustering algorithm is tested on six datasets from UCI Machine Learning Repository [37] and the obtained results are compared with those obtained using other similar algorithms.

The rest of this paper is organized as follows.  Section 2 describes clustering problem. A brief overview of the BBO algorithm is given in Section 3.  Section 4 presents the clustering algorithm. Experimental results are reported in Section 5. Finally, Section 6 presents conclusions with future research direction.

## 2. Cluster Analysis

In cluster analysis we suppose that we have been given a set *A* of a finite number of points of *d*-dimensional space *R* ^*d*^, that is {*a* ^1^, *a* ^2^,…, *a* ^*n*^}, where *a* ^*i*^ ∈ *R* ^*d*^,   *i* = 1,2,…, *n*.

In all, clustering algorithms can be classified into two categories, namely, hierarchical clustering and partitional clustering. Partitional clustering methods are the most popular class of center based clustering methods. It has been seen that partitional algorithm is more commendable rather than hierarchical clustering. The advantage of partitional algorithm is its visibility in circumstances where application involving large dataset is used where construction of nested grouping of patterns is computationally prohibited [38, 39]. The clustering problem is said to be hard clustering if every data point belongs to only one cluster. Unlike hard clustering, in the fuzzy clustering problem the clusters are allowed to overlap and instances have degrees of appearance in each cluster [40]. In this paper we will exclusively consider the hard unconstrained clustering problem. Therefore, the subject of cluster analysis is the partition of the set *A* into a given number *q* or disjoint subsets *B*_*i*_,   *i* = 1,2,…, *q*, with respect to predefined criteria such that

(1) Bi≠∅,i=1,2,…,q,Bi∩Bj=∅,∀i≠j,  i,j=1,2,…,q,⋃i=1qBi=A.

Each cluster *B*_*i*_ can be identified by its center (or centroid). To determine the dissimilarity between objects, many distance metrics have been defined. The most popular distance metric is the Euclidean distance. In this research we will also use Euclidean metric as a distance metric to measure the dissimilarity between data objects. So, for given two objects *a* ^*i*^ and *a* ^*j*^ with *d*-dimensions, the distance is defined by [38] as

(2) $$\begin{array}{c} d\left(\;a^{i},a^{j}\;\right)=\sqrt{\overset{d}{\underset{r=1}{\sum }}{\left(a_{i}^{r}-a_{j}^{r}\right)}^{2}}. \end{array}$$

Since there are different ways to cluster a given set of objects, a fitness function (cost function) for measuring the goodness of clustering should be defined. A famous and widely used function for this purpose is the total mean-square quantization error (MSE) [41], which is defined as follows:

(3) $$\begin{array}{c} MSE=\overset{q}{\underset{j=1}{\sum }}\;\underset{a^{i}\in B_{j}}{\sum }d{\left(\;a^{i},B_{j}\;\right)}^{2}, \end{array}$$

where *d*(*a* ^*i*^, *B*_*j*_)^2^ is the distance between object *a* ^*i*^ and the center of cluster *C*_*j*_(*B*_*j*_) to be found by calculating the mean value of objects within the respective cluster.

## 3. Biogeography-Based Optimization Algorithm

In this section, we give a brief description of the Biogeography-Based Optimization (BBO) algorithm. BBO is a new evolutionary optimization method based on the study of geographic distribution of biological organisms (biogeography) [34]. Organisms in BBO are called species, and their distribution is considered over time and space. Species can migrate between islands which are called habitat. Habitat is characterized by a Habitat Suitability Index (HSI). HSI in BBO is similar to the fitness in other population-based optimization algorithms and measures the solution goodness. HSI is related to many features of the habitat [34]. Considering a global optimization problem and a population of candidate solutions (individuals), each individual can be considered as a habitat and is characterized by its HSI. A habitat with high HSI is a good solution (maximization problem). Similar to other evolutionary algorithms, good solutions share their features with others to produce a better population in the next generations. Conversely, an individual with low fitness is unlikely to share features and likely accept features. Suitability index variable (SIV) implies the habitability of a habitat. As there are many factors in the real world which make a habitat more suitable to reside than others, there are several SIVs for a solution which affect its goodness. A SIV is a feature of the solution and can be imagined like a gene in GA. BBO consists of two main steps: migration and mutation. Migration is a probabilistic operator that is intended to improve a candidate solution [42, 43]. In BBO, the migration operator includes two different types: immigration and emigration, where for each solution in each generation, the rates of these types are adaptively determined based on the fitness of the solution. In BBO, each candidate solution *h*_*i*_ has its own immigration rate *λ*_*i*_ and emigration rate *μ*_*i*_ as follows:

(4) λi=I1−kinpop,μi=Ekinpop,

where *n*pop is the population size and *k*(*i*) shows the rank of *i*th individual in a ranked list which has been sorted based on the fitness of the population from the worst fitness to the best one (1 is worst and *n*pop is best). Also *E* and *I* are the maximum possible emigration and immigration rates, which are typically set to one. A good candidate solution has latively high emigration rate and allows immigration rate, while the converse is true for a poor candidate solution. Therefore if a given solution *h*_*i*_ is selected to be modified (in migration step), then its immigration rate *λ*_*i*_ is applied to probabilistically modify each SIV in that solution. The emigrating candidate solution *h*_*j*_ is probabilistically chosen based on *μ*_*j*_. Different methods have been suggested for sharing information between habitats (candidate solutions), in [44], where migration is defined by

(5) $$\begin{array}{c} h_{i}\left(\;SIV\;\right)=\alpha {*h}_{i}\left(\;SIV\;\right)+\left(\;1-\alpha \;\right)*h_{j}\left(\;SIV\;\right), \end{array}$$

where *α* is a number between 0 and 1. It could be random or deterministic or it could be proportional to the relative fitness of the solutions *h*_*i*_ and *h*_*j*_. Equation (5) means that (feature solution) SIV of *h*_*i*_ comes from a combination of its own SIV and the emigrating solution's SIV. Mutation is a probabilistic operator that randomly modifies a decision variable of a candidate solution. The purpose of mutation is to increase diversity among the population. The mutation rate is calculated in [34]

(6) $$\begin{array}{c} m_{i}=m_{max}\left(\;\frac{1-P_{i}}{P_{max}}\;\right), \end{array}$$

where *P*_*i*_ is the solution probability and  *P*_max_ = max_*i*_⁡*P*_*i*_, *i* = 1,…, *n*pop, where *n*pop is the population size and *m*_max_ is user-defined parameter.

If *h*_*i*_(SIV) is selected for mutation, then the candidate solution *h*_*j*_ is probabilistically chosen based on *m*_*i*_; thus replace *h*_*i*_(SIV) with a randomly generated SIV. Several options can be used for mutation but one option for implementing that can be defined as

(7) $$\begin{array}{c} h_{i}\left(\;SIV\;\right)=h_{i}\left(\;SIV\;\right)+\rho , \end{array}$$

where

(8) $$\begin{array}{c} \rho =\partial \left(\;\max\left(\;h_{i}\left(\;SIV\;\right)\;\right)-\min\left(\;h_{i}\left(\;SIV\;\right)\;\right)\;\right)\sigma . \end{array}$$

∂ is user-defined parameter near 0 and also max⁡(*h*_*i*_(SIV)),  min⁡(*h*_*i*_(SIV)) are the upper and lower bounds for each decision variable and *σ* is random number, normally distributed in the range of (0, 1).

Based on the above description, the main steps of the BBO algorithm can be described as follows.

**Step 1 (initialization).**

At first, introduce the initial parameters that include the number of generations, necessary for the termination criterion, population size, which indicates the number of habitats/islands/solutions, number of design variables, maximum immigration and emigration rates, and mutation coefficient and also create a random set of habitats (population).

**Step 2 (evaluation).**

Compute corresponding HSI values and rank them on the basis of fitness.

**Step 3 (update parameters).**

Update the immigration rate *λ*_*i*_ and emigration rate *μ*_*i*_ for each island/solution. Bad solutions have low emigration rates and high immigration rates whereas good solutions have high emigration rates and low immigration rates.

**Step 4 (select islands).**

Probabilistically select the immigration islands based on the immigration rates and select the emigrating islands based on the emigration rates via roulette wheel selection.

**Step 5 (migration phase).**

Randomly change the selected features (SIVs), based on (4)–(5) and based on the selected islands in the previous step.

**Step 6 (mutation phase).**

Probabilistically carry out mutation based on the mutation probability for each solution, that is, based on (6).

**Step 7 (check the termination criteria).**

If the output of the termination criterion step is not met, go to Step 2; otherwise, terminate it.

## 4. BBO Algorithm for Data Clustering

**Figure 1:**
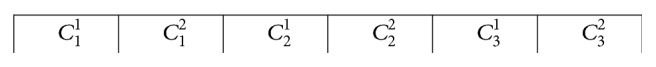
The encoding of an example of candidate solution.

In order to use BBO algorithm for data clustering, one-dimensional arrays are used to encode the centres of the desired clusters to present candidate solutions in the proposed algorithm. The length of the arrays is equal to *q* × *d*, where *q* is the number of clusters and *d* is the dimensionality of the considered datasets.  Figure 1 presents an example of candidate solution for a problem with 3 centroids clusters and 2 attributes.

Then assume POP_*i*_ = {*C*_1_, *C*_2_,…, *C*_*q*_} is the *i*th candidate solution and  *C*_*j*_ = {*C*_*j*_^1^, *C*_*j*_^2^,…, *C*_*j*_^*d*^} is the *j*th cluster centre for the *i*th candidate solution (*i* = 1,2,…, *n*pop) and (*j* = 1,2,…, *q*), so that *n*pop is the number of islands or candidate solutions in which its value in this work is set to 100. Therefore each of these candidate solutions shows centers of all clusters.

A good initial population is important to the performance of BBO and most of the population-based methods are affected by the quality of the initial population. Then in the proposed algorithm, taking into considering the nature of the input datasets, a high-quality population is created based on special ways as mentioned in pseudocodes. One of the candidate solutions will be produced by dividing whole dataset to *q* equal sets, and three of them will be produced based on minimum, maximum, and average values of data objects in each dataset and other solutions will be created randomly. This procedure creates a high-quality initial population and consequently this procedure ensures that the candidate solutions are spread in the wide area of the search space, which as a result increases the chance of finding (near) global optima.

To ensure that the best habitats/solutions are preserved, elitist method is used to save the best individual found so far into the new population. So elitism strategy is proposed in order to retain the best solutions in the population from one generation to the next. Therefore in the proposed algorithm, new population is created based on merging initial population (old population) and the population due to migration and mutation process (new population). Then suppose POP is the entire initial population of candidate solutions and *New* *POP* is the initial population, changed by iteration of BBO, and *γ* is percentage of initial population that is chosen in next iteration (whose value in this work is 30%). So the number of kept habitats of old population (KHOP) is as follows:

(9) $$\begin{array}{c} KHOP=round\left(\;\gamma \times npop\;\right). \end{array}$$

And the number of kept habitats of new population (KHCP) is as follows:

(10) $$\begin{array}{c} KHCP=npop-KHOP. \end{array}$$

Hence the population of next iteration can be as follows:

(11) $$\begin{array}{c} POP⟵\left[\begin{array}{c} POP\left(1\text{:}\;KHOP\right)\\ NewPOP\left(1\text{:}\;KHCP\right) \end{array}\right]. \end{array}$$

Suppose POP_*i*_ is the *i*th candidate solution and POP_*i*_(*s*) is the *s*th decision variable of POP_*i*_  (i.e.  *C*_*r*_^*t*^, *t* = 1,2,…, *d*  and  *r* = 1,2,…, *q*). Based on the above description, the pseudocode of the proposed method is shown in Algorithm 1.

## 5. Experimental Results

The proposed method is implemented using MATLAB 7.6 on a T6400, 2 GHz, 2 GB RAM computer. To evaluate the performance of the proposed algorithm, the results obtained have been compared with other algorithms by applying them on some well known datasets taken from Machine Learning Laboratory [37]. Six datasets are employed to validate the proposed method. These datasets named Cancer, CMC, Iris, Glass, Wine, and Vowel cover examples of data of low, medium, and high dimensions. The brief of the characteristics of these datasets is presented in Table 1. They have been applied by many authors to study and evaluate the performance of their algorithms, and they can be described as follows.

*Wisconsin Breast Cancer Dataset *(*n* = 683, *d* = 9,   *k* = 2). This dataset has* 683* points with nine features such as cell size uniformity, clump thickness cell, bare nuclei, shape uniformity, marginal adhesion, single epithelial cell size, bland chromatin, normal nucleoli, and mitoses. There are two clusters in this dataset: malignant and benign. 

*Contraceptive Method Choice Dataset *(*denoted* *as* *CMC* *with* *n* = 1473, *d* = 10,   *k* = 3). This dataset is a subset of the 1987 National Indonesia Contraceptive Prevalence Survey. The samples are married women who either were not pregnant or did not know if they were at the time of interview. The problem is to predict the choice of current contraceptive method (no use has 629 objects, long-term methods have* 334 *objects, and short-term methods have* 510 *objects) of a woman based on her demographic and socioeconomic characteristics. 

*Ripley's Glass Dataset *(*n* = 214,   *d* = 9,   *k* = 6). This dataset has* 214* points with nine features. The dataset has six different clusters which are building windows float processed, building windows nonfloat processed, vehicle windows float processed, containers, tableware, and headlamps [41]. 

*Iris Dataset *(*n* = 150, *d* = 4,   *k* = 3). This data consists of three different species of iris flower:* Iris setosa*,* Iris virginica*, and* Iris versicolour*. For each species, 50 samples with four features each (sepal length, sepal width, petal length, and petal width) were collected [45]. 

*Vowel Dataset *(*n* = 871, *d* = 3,   *k* = 6). It consists of* 871 *Indian Telugu vowel sounds. The dataset has three features corresponding to the first, second, and third vowel frequencies and six overlapping classes [45]. 

*Wine Dataset *(*n* = 178,   *d* = 13, *k* = 3). This dataset describes the quality of wine from physicochemical properties in Italy. There are* 178* instances with* 13 *continues attributes grouped into 3 classes. There is no missing value for attributes.

In this paper the performance of the proposed algorithm is compared with recent algorithms reported in the literature, including *K*-means [38], TS [19], SA [20], PSO [22, 39], BB-BC [27], GA [21], GSA [30], and ACO [46].

In this paper two criteria are used to measure the quality of solutions found by clustering algorithms:

(i) *Sum of intracluster distances:* The distance between each data vector in a cluster and the centroid of that cluster is calculated and summed up, as defined in (3). It is also the evaluation fitness in this paper. Clearly, the smaller the value is, the higher the quality of the clustering is.
(ii) *Error rate (ER):* It is defined as the number of misplaced points over the total number of points in the dataset as
  (12) $$\begin{array}{c} ER=\frac{\left(\sum_{i=1}^{npop}\left(\text{if}\;\;\left(B_{i}=C_{i}\right)\text{ then }0\text{ else }1\right)\right)}{npop}*100, \end{array}$$

where *n*pop is the total number of data points and *B*_*i*_ and *C*_*i*_ denote the datasets of which the *i*th point is a member before and after clustering, respectively.

Since all the algorithms are stochastic algorithms, therefore for each experiment 10 independent runs are carried out to indicate the stability and robustness of the algorithms for against with the randomized nature of the algorithms. The average, best (minimum), and worst (maximum) solutions and standard deviation of solutions of 10 runs of each algorithm are obtained by using algorithms on the datasets, which have been applied for comparison. This process ensures that the candidate solutions are spread in the wide area of the search space and thus increases the chance of finding optima.

Table 2 presents the intracluster distances obtained from the eight clustering algorithms for the datasets above. For the* cancer* dataset, the average, best, and worst solutions of BBO algorithm are 2964.3879, 2964.3875, and 2964.3887, respectively, which are much better than those of other algorithms except BB-BC which is the same as it. This means that it provides the optimum value and small standard deviation, when compared to those obtained by the other methods. For the CMC dataset, the proposed method reaches an average of 5532.2550, while other algorithms were unable to reach this solution. Also, the results obtained on the* glass* dataset show that BBO method converges to the optimum of 215.2097 in all of runs while the average solutions of the *k*-means, TS, SA, GA, PSO, BB-BC, GSA, and ACO, are 227.9779, 283.79, 282.19, 230.49328, 231.2306, 255.38, 233.5433, and 273.46, respectively. For the* iris* dataset, the average of solutions found by BBO is 96.5653, while this value for the *k*-means, TS, SA, GA, PSO, BB-BC, GSA, and ACO, is 105.7290, 97.8680, 99.95, 98.1423, 96.7654, 125.1970, 96.7311, and 97.1715, respectively. As seen from the results for the* vowel* dataset, the BBO algorithm outperformed the *K*-means, TS, SA, GA, PSO, BB-BC, GSA, and ACO algorithms, with the average solution 149072.9042. For the* Wine* dataset, the BBO algorithm achieved the optimum value of 16292.6782, which is significantly better than the other tested algorithms.

From Table 2, we can see that the BBO algorithm has achieved the good performance in terms of the average, best, and worst intercluster distances on these six datasets. It means that BBO can find good quality solutions.

The best centroids coordinates obtained by the BBO algorithm on the test dataset are shown in Tables 3 [Table tab4] [Table tab5] [Table tab6] [Table tab7]–8. Finally, Table 9 shows the error rate values obtained by algorithms for real datasets. As seen from the results in Table 9, the BBO algorithm presents a minimum average error rate in all the real datasets. However, the topography of the cost function of clustering (3) has a valley shape; therefore the found solutions by these methods were not global. Therefore the experimental results in the tables demonstrate that the proposed method is one of practicable and good techniques for data clustering.

## 6. Conclusions

In summary, this paper presents a new clustering algorithm based on the recently developed BBO heuristic algorithm that is inspired by mathematical models of science of biogeography (study of the distribution of animals and plants over time and space).

To evaluate the performance of the BBO algorithm, it was tested on six real life datasets and compared with other eight clustering algorithms. The experimental results indicate that the BBO optimization algorithm is suitable and useful heuristic technique for data clustering. In order to improve the obtained results, as a future work, we plan to hybridize the proposed approach with other algorithms and we intend to apply this method with other data mining problems.

## Figures and Tables

**Algorithm 1:**
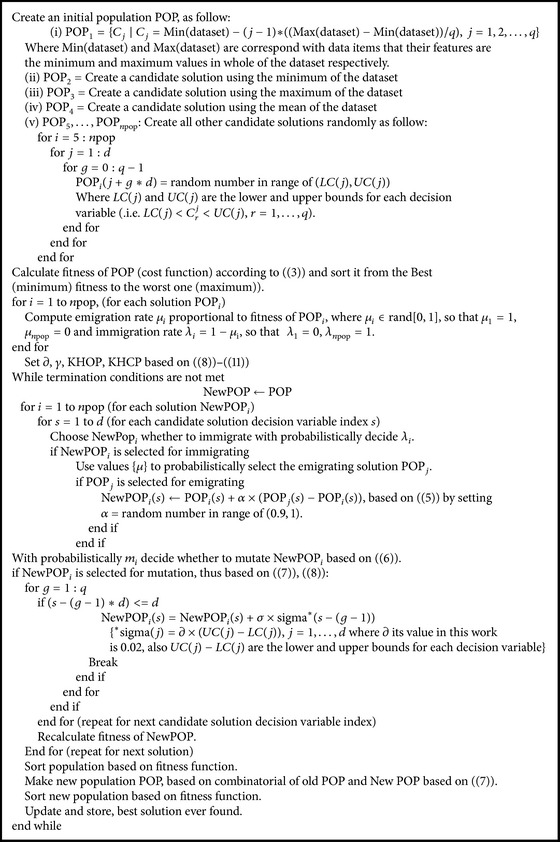
Pseudocodes of proposed method.

**Table 1:** Summarized characteristics of the test datasets.

Name of dataset	Number of data objects	Number of features	Number of clusters
Cancer	683	9	2 (444, 239)
CMC	1473	9	3 (629, 334, 510)
Glass	214	9	6 (70, 76, 17, 13, 9, 29)
Iris	150	4	3 (50, 50, 50)
Vowel	871	3	6 (72, 89, 172, 151, 207, 180)
Wine	178	13	3 (59, 71, 48)

**Table 2:** Intracluster distances for real life datasets.

Dataset	Criteria	*K*-means	TS	SA	PSO	BB-BC	GA	GSA	ACO	BBO
Cancer	Average	3032.2478	3251.37	3239.17	2981.7865	2964.3880	3249.46	2972.6631	3,046.06	2964.3879
	Best	2986.9613	2982.84	2993.45	2974.4809	2964.3875	2999.32	2965.7639	2,970.49	2964.3875
	Worst	5216.0895	3434.16	3421.95	3053.4913	2964.3890	3427.43	2993.2446	3,242.01	2964.3887
	Std.	315.1456	232.217	230.192	10.43651	0.00048	229.734	8.91860	90.50028	0.00036

CMC	Average	5543.4234	5993.59	5893.48	5547.8932	5574.7517	5756.59	5581.9450	5,819.1347	5532.2550
	Best	5542.1821	5885.06	5849.03	5539.1745	5534.0948	5705.63	5542.2763	5,701.9230	5532.2113
	Worst	5545.3333	5999.80	5966.94	5561.6549	5644.7026	5812.64	5658.7629	5,912.4300	5532.432
	Std.	1.5238	40.845	50.867	7.35617	39.4349	50.369	41.13648	45.634700	0.06480

Glass	Average	227.9779	283.79	282.19	230.49328	231.2306	255.38	233.5433	273.46	215.2097
	Best	215.6775	279.87	275.16	223.90546	223.8941	235.50	224.9841	269.72	210.6173
	Worst	260.8385	286.47	287.18	246.08915	243.2088	278.37	248.3672	280.08	233.9314
	Std.	14.1389	4.19	4.238	4.79320	4.6501	12.47	6.13946	3.5848	3.525

Iris	Average	105.7290	97.8680	99.95	98.1423	96.7654	125.1970	96.7311	97.1715	96.5653
	Best	97.3259	97.3659	97.45	96.8793	96.6765	113.9865	96.6879	97.1007	96.5403
	Worst	128.4042	98.56949	102.01	99.7695	97.4287	139.7782	96.8246	97.8084	96.6609
	Std.	12.3876	72.86	2.018	0.84207	0.20456	14.563	0.02761	0.367	0.0394

Vowel	Average	153,660.8071	162108.53	161566.28	153,218.23418	151,010.0339	159153.49	152,931.8104	159,458.1438	149072.9042
	Best	149,394.8040	149468.26	149370.47	152,461.56473	149,038.5168	149513.73	151,317.5639	149,395.602	148967.2544
	Worst	168,474.2659	165996.42	165986.42	158,987.08231	153,090.4407	165991.65	155,346.6952	165,939.8260	153051.96931
	Std.	4123.04203	2846.235	0.645	2945.23167	1859.3235	3105.544	2486.70285	3,485.3816	137.7311

Wine	Average	16,963.0441	16785.46	17,521.09	16,316.2745	16,303.4121	16,530.53	16,374.3091	16,530.53381	16292.6782
	Best	16,555.6794	16666.22	16,473.48	16,304.4858	16,298.6736	16,530.53	16,313.8762	16,530.53381	16292.6782
	Worst	23,755.0495	16837.54	18,083.25	16,342.7811	16,310.1135	16,530.53	16,428.8649	16,530.53381	16292.6782
	Std.	1180.6942	52.073	753.084	12.60275	2.6620	0	34.67122	0	0

**Table 3:** The obtained best centroids coordinate for *Cancer* data.

Cancer data	Cluster 1	Cluster 2
Feature A	7.1156	2.8896
Feature B	6.6398	1.1278
Feature C	6.6238	1.2018
Feature D	5.6135	1.1646
Feature E	5.2402	1.9943
Feature F	8.0995	1.1215
Feature G	6.0789	2.0059
Feature H	6.0198	1.1014
Feature I	2.3282	1.0320

**Table 4 tab4:** The obtained best centroids coordinate for *CMC* data.

CMC data	Cluster 1	Cluster 2	Cluster 3
Feature A	43.6354	33.4957	24.4102
Feature B	3.0140	3.1307	3.0417
Feature C	3.4513	3.5542	3.5181
Feature D	4.582	3.6511	1.7947
Feature E	0.7965	0.7928	0.9275
Feature F	0.7629	0.6918	0.7928
Feature G	1.8245	2.0903	2.2980
Feature H	3.4355	3.29183	2.9754
Feature I	0.094	0.0573	0.037

**Table 5 tab5:** The obtained best centroids coordinate for *Glass *data.

Glass data	Cluster 1	Cluster 2	Cluster 3	Cluster 4	Cluster 5	Cluster 6
Feature A	1.5260	1.5156	1.5228	1.5266	1.5203	1.5243
Feature B	11.9759	13.0863	14.6577	13.2229	13.7277	13.8085
Feature C	0.006	3.5272	0.0061	0.4232	3.5127	2.3414
Feature D	1.0514	1.3618	2.2170	1.5242	1.0249	2.5919
Feature E	72.0540	72.8710	73.2504	73.0610	71.9072	71.1423
Feature F	0.2552	0.5768	0.0299	0.3865	0.2067	2.5749
Feature G	14.3566	8.3588	8.6714	11.1471	9.4166	5.9948
Feature H	0.1808	0.0046	1.047	0.00979	0.0281	1.3373
Feature I	0.1254	0.0568	0.0196	0.1544	0.0498	0.2846

**Table 6 tab6:** The obtained best centroids coordinate for *Iris* data.

Iris data	Cluster 1	Cluster 2	Cluster 3
Feature A	5.0150	5.9338	6.7343
Feature B	3.4185	2.7974	3.0681
Feature C	1.4681	4.4173	5.6299
Feature D	0.2380	1.4165	2.1072

**Table 7 tab7:** The obtained best centroids coordinate for *Vowel *data.

Vowel data	Cluster 1	Cluster 2	Cluster 3	Cluster 4	Cluster 5	Cluster 6
Feature A	357.8349	375.8459	508.1135	407.9219	623.6778	439.6126
Feature B	2,291.6435	2,148.4110	1,838.2133	1,0182.0145	1,309.8038	987.4300
Feature C	2,978.2399	2,678.8524	2,555.9085	2,317.2847	2,332.7767	2,665.4154

**Table 8:** The obtained best centroids coordinates for *Wine* data.

Wine data	Cluster 1	Cluster 2	Cluster 3
Feature A	13.3856	12.7859	12.7093
Feature B	1.9976	2.3535	2.3219
Feature C	2.3150	2.4954	2.4497
Feature D	16.9836	19.5480	21.1983
Feature E	105.2124	98.9327	92.6449
Feature F	3.0255	2.0964	2.1366
Feature G	3.1380	1.4428	1.9187
Feature H	0.51050	0.31322	0.3520
Feature I	2.3769	1.7629	1.4966
Feature J	5.7760	5.8415	4.3213
Feature K	0.8339	1.1220	1.2229
Feature L	3.0686	1.9611	2.5417
Feature M	1137.4923	687.3041	463.8856

**Table 9:** Error rates for real life datasets.

Dataset	*K*-means	PSO	GSA	BBO
Cancer	4.08	5.11	3.74	3.7
CMC	54.49	54.41	55.67	54.22
Glass	37.71	45.59	41.39	36.47
Iris	17.80	12.53	10.04	10.03
Vowel	44.26	44.65	42.26	41.36
Wine	31.12	28.71	29.15	28.65
